# Dissimilatory Fe(III) Reduction Controls on Arsenic Mobilization: A Combined Biogeochemical and NanoSIMS Imaging Approach

**DOI:** 10.3389/fmicb.2021.640734

**Published:** 2021-02-22

**Authors:** Rebeca Lopez-Adams, Laura Newsome, Katie L. Moore, Ian C. Lyon, Jonathan R. Lloyd

**Affiliations:** ^1^Department of Earth and Environmental Sciences, University of Manchester, Manchester, United Kingdom; ^2^Camborne School of Mines, Environment and Sustainability Institute, University of Exeter, Exeter, United Kingdom; ^3^Department of Materials, University of Manchester, Manchester, United Kingdom; ^4^Photon Science Institute, University of Manchester, Manchester, United Kingdom

**Keywords:** As(V), As(III), iron(III)-oxyhydroxide, flavins, extracellular electron transfer, nanoSIMS, *Shewanella*, dissimilatory Fe(III) reduction

## Abstract

Microbial metabolism plays a key role in controlling the fate of toxic groundwater contaminants, such as arsenic. Dissimilatory metal reduction catalyzed by subsurface bacteria can facilitate the mobilization of arsenic *via* the reductive dissolution of As(V)-bearing Fe(III) mineral assemblages. The mobility of liberated As(V) can then be amplified *via* reduction to the more soluble As(III) by As(V)-respiring bacteria. This investigation focused on the reductive dissolution of As(V) sorbed onto Fe(III)-(oxyhydr)oxide by model Fe(III)- and As(V)-reducing bacteria, to elucidate the mechanisms underpinning these processes at the single-cell scale. Axenic cultures of *Shewanella* sp. ANA-3 wild-type (WT) cells [able to respire both Fe(III) and As(V)] were grown using ^13^C-labeled lactate on an arsenical Fe(III)-(oxyhydr)oxide thin film, and after colonization, the distribution of Fe and As in the solid phase was assessed using nanoscale secondary ion mass spectrometry (NanoSIMS), complemented with aqueous geochemistry analyses. Parallel experiments were conducted using an *arrA* mutant, able to respire Fe(III) but not As(V). NanoSIMS imaging showed that most metabolically active cells were not in direct contact with the Fe(III) mineral. Flavins were released by both strains, suggesting that these cell-secreted electron shuttles mediated extracellular Fe(III)-(oxyhydr)oxide reduction, but did not facilitate extracellular As(V) reduction, demonstrated by the presence of flavins yet lack of As(III) in the supernatants of the *arr*A deletion mutant strain. 3D reconstructions of NanoSIMS depth-profiled single cells revealed that As and Fe were associated with the cell surface in the WT cells, whereas for the *arr*A mutant, only Fe was associated with the biomass. These data were consistent with *Shewanella* sp. ANA-3 respiring As(V) in a multistep process; first, the reductive dissolution of the Fe(III) mineral released As(V), and once in solution, As(V) was respired by the cells to As(III). As well as highlighting Fe(III) reduction as the primary release mechanism for arsenic, our data also identified unexpected cellular As(III) retention mechanisms that require further investigation.

## Introduction

In environments where oxygen is absent or depleted, bacteria and archaea are able to use a wide range of alternative terminal electron acceptors to conserve energy (Weber et al., 2006). Fe(III) respiration by dissimilatory metal-reducing bacteria is a well-known example of these anaerobic processes (Lovley and Phillips, 1988; Lee and Newman, 2003; Lloyd, 2003). Physicochemical conditions dictate the chemical form in which iron is found in the environment, with Fe(II) and Fe(III) most common (Hedrich et al., 2011). In anoxic conditions, ferrous iron [Fe(II)] predominates, whereas in oxygen-rich environments, ferric iron [Fe(III)] is the predominant species and forms Fe(III) minerals at circumneutral pH. As these Fe(III) minerals are usually poorly soluble, this poses the challenge of transferring electrons to the cell surface *via* an extracellular electron transport system when respiring Fe(III) (Nevin and Lovley, 2002; Lovley et al., 2004; Reguera et al., 2005; Weber et al., 2006).

Fe(III) minerals are strong sorbents of metals and metalloids, such as arsenic (Pierce and Moore, 1982; Belzile and Tessier, 1990), and toxic sorbed metals are known to be released to the solution during the reductive dissolution of Fe(III) minerals by Fe(III)-reducing bacteria (Sullivan and Aller, 1996; Nickson et al., 2000; Islam et al., 2004; Revesz et al., 2015, 2016). Arsenic is a toxic element that occurs naturally in rocks, sediments and groundwater; its concentration and incidence in soils and freshwaters greatly depend on host geology, but also on anthropological contributions. For example, levels can be increased through mining or pesticide use in agriculture (Ahmann et al., 1997; Bowell et al., 2014). The environmental behavior of arsenic is influenced strongly by iron mineral transformations and by redox geochemistry. The most common forms of arsenic are inorganic trivalent arsenite [As(III)] and pentavalent arsenate [As(V)] oxyanions (Cullen and Reimer, 1989; Bowell et al., 2014). As(V) and As(III) have different sorption behaviors to iron mineral surfaces (Tufano et al., 2008). Fe(III) minerals (e.g., ferrihydrite, goethite, and hematite) have the capability to sorb, sequester and precipitate both As species, although As(III) is sorbed through weakly-bound complexes, desorbing more easily than As(V) (Tufano et al., 2008). Therefore, As(III) is regarded as the more soluble and mobile of these oxyanions and is also considered to be more toxic than As(V) (Cullen and Reimer, 1989; Shen et al., 2013). Anions, such as phosphate, promote As(V) desorption by competing for sorption sites in the subsurface, for instance, on Fe(III)-(oxyhydr)oxide minerals (Biswas et al., 2014). Desorbed As(V) is more bioavailable than sorbed As(V); therefore, phosphate-promoted As(V) desorption and As(V) release *via* the reductive dissolution of Fe(III)-(oyhydr)oxides are critical steps in As mobilization in the environment (Biswas et al., 2014; Glasser et al., 2018). Additionally, field studies in As-impacted aquifers and lakes have shown that As(V) predominates near the surface, whereas As(III) becomes dominant with increasing depth (Oremland and Stolz, 2003; Gnanaprakasam et al., 2017; Podgorski et al., 2017). The occurrence of As(V) in the solid phase usually correlates with the presence of amorphous Fe(III) minerals, which are subject to reductive dissolution and transformation as depth increases, depleting the available As sorption sites and promoting As(V) desorption (Tufano et al., 2008; Gnanaprakasam et al., 2017).

In the subsurface, As can be mobilized through multiple mechanisms, linked to a diversity of bacterial metabolisms (Zobrist et al., 2000). For example, (i) Fe(III)-respiring bacteria could desorb As(V) *via* the reductive dissolution of Fe(III) minerals, (ii) dissimilatory As(V)-reducing bacteria could directly reduce sorbed As(V) to poorly sorbing As(III) or (iii) As could be mobilized by a sequential process where dissimilatory Fe(III) and As(V)-respiring bacteria first desorb As(V) from Fe(III) minerals *via* Fe(III) reduction and then respire the soluble As(V) (Zobrist et al., 2000; Tufano et al., 2008; Huang et al., 2011; Tian et al., 2015; Lin et al., 2019). The exact mechanisms leading to As(V) release, reduction and accumulation of As(III) still remain unclear and are the subject of investigation in this study.

*Shewanella* is a genus of metabolically flexible facultative anaerobic bacteria (Hau and Gralnick, 2007; Fredrickson et al., 2008). The four proposed mechanisms for the reduction of poorly soluble extracellular Fe(III)-(oxyhydr)oxide minerals by *Shewanella* species are: (i) use of an endogenous (e.g., flavin) or exogenous (e.g., humic acid) electron shuttle (Nevin and Lovley, 2002), (ii) using chelators to mobilize Fe(III) for intracellular reduction (Lovley, 1991), (iii) *via* the direct contact of solid Fe(III) minerals with outer membrane heme-containing *c*-type cytochromes (Gorby et al., 2006; Weber et al., 2006) and (iv) by using electrically conductive nanowires in the form of outer membrane protein extensions, analogous to nanowires in *Geobacter* (Malvankar et al., 2014; Pirbadian et al., 2014; Adhikari et al., 2016; Reguera, 2018; Yalcin and Malvankar, 2020), although this mechanism has been challenged (Subramanian et al., 2018). These mechanisms contrast with other Fe(III)-reducing species, such as *Geobacter* spp., which require direct contact with Fe(III) minerals to facilitate extracellular electron transport in the absence of an exogenous electron shuttle (Nevin and Lovley, 2000; Childers et al., 2002). Flavin mononucleotide (FMN) and riboflavin have been identified as the electron shuttles secreted by different *Shewanella* strains (von Canstein et al., 2008). Moreover, when *Shewanella oneidensis* MR-1 is grown in biofilms using electrodes as terminal electron acceptors, flavins have been shown to bind to outer membrane cytochromes, forming flavocytochrome complexes that accelerate extracellular electron transfer (Okamoto et al., 2013; Edwards et al., 2015; Xu et al., 2016).

The mechanism of electron flow from organic electron donors (such as lactate, pyruvate or amino acids) in *Shewanella* cells involves the Mtr pathway, where six multihaem *c*-type cytochromes (CymA, Fcc_3_, MtrA, MtrC, OmcA, and STC) and a porin-like outer membrane protein (MtrB) transport electrons from the menaquinone pool in the cytoplasmic membrane to the cell surface (Beblawy et al., 2018). Electrons are carried from the cell surface *via* flavins until they reach the surface of an insoluble electron acceptor (Shi et al., 2012, 2016).

In contrast to Fe(III) mineral reduction, As(V) reduction is not mediated *via* the complex network of *c*-type cytochromes present in Fe(III)-reducing bacteria. Moreover, it is not clear if flavins, as electron shuttling compounds, could promote As(V) reduction, in addition to their As(III) oxidizing capabilities (Pi et al., 2019). In most cases, this reduction, which is widespread in bacteria and yeast (Cullen and Reimer, 1989; Oremland and Stolz, 2003), is part of a resistance or detoxification process and is mediated through the well-studied intracellular ArsC As(V) reductase system (Oremland and Stolz, 2003; Saltikov et al., 2003; Héry et al., 2008), *via* a process that uses energy, rather than conserving it for growth. A restricted group of microorganisms, the dissimilatory As(V)-reducing bacteria, can, however, reduce As(V) to conserve energy, through the periplasmic *arr* reductase system (Oremland and Stolz, 2003). *Shewanella* sp. strain ANA-3 possesses both the As(V) detoxification and respiratory systems (Saltikov and Newman, 2003).

Nanoscale secondary ion mass spectrometry (NanoSIMS) is a high-resolution imaging technique that allows the identification of metabolically active microorganisms, linking function and identity, and the semi-quantification of trace element abundances (Li et al., 2008). The high spatial resolution of NanoSIMS, down to 50 nm, makes this technique suitable to study microbial cells, even at the subcellular level (Hoppe et al., 2013). For instance, NanoSIMS has been applied to study cyanobacteria within hypersaline microbial mats (Fike et al., 2008), to study phototrophic bacteria catalyzing C and N cycling in Lake Cadagno sediments (Musat et al., 2008), to study methane-oxidizing microbial consortia to elucidate syntrophy *via* electron transfer between archaea and bacteria (McGlynn et al., 2015), to infer distinct extracellular electron transport mechanisms in Fe(III)-respiring bacteria (Newsome et al., 2018) and to reveal the stratified metabolic activity of *Geobacter sulfurreducens* cells growing in biofilms influenced by their distance to the electrode surface (Chadwick et al., 2019).

The aim of this work was to study the reduction of As(V) sorbed onto an Fe(III)-(oxyhydr)oxide by two *Shewanella* sp. ANA-3 strains using NanoSIMS. The technique was used to measure bulk and trace concentrations of As and Fe, by (1) imaging the mineral surface and (2) by generating single-cell depth profiles to allow the subcellular localization of these two elements to be determined. To investigate the mechanism of As release, particularly the impact of As(V) respiration, studies were performed with the *S*. ANA-3 wild-type (WT) strain and the *S*. ANA-3 *arr*A deletion mutant. In these conditions, As(V) respiration to As(III) is expected to be a key process controlling As mobilization, in accordance with previous studies (Tufano et al., 2008; Huang et al., 2011; Lin et al., 2019). The effect of flavins on As(V) reduction, as an example of secreted electron shuttles and redox-active compounds, was also assessed in this work. This approach using NanoSIMS to study the fate of As in the solid phase, together with complementary aqueous geochemical techniques, has advanced our understanding of the mechanisms that lead to the mobilization of As(III) in anoxic subsurface environments.

## Materials and Methods

### Bacterial Strains and Culture

*Shewanella* sp. ANA-3 strain WT and the non-As(V)-respiring *arr*A deletion mutant, *S.* ANA-3 strain ARRA3, were used in these experiments. The bacterial strains were kindly donated by Professor Dianne Newman (California Institute of Technology). Both strains were grown on aerobic lysogeny broth (LB) agar plates at 30°C for 24 h. Isolated colonies were used to inoculate aerobic LB in Erlenmeyer flasks and incubated at 30°C/150 rpm for 24 h until late-logarithmic phase. Five ml of this aerobic culture was transferred to inoculate serum bottles containing 100 ml of anoxic minimal growth medium [in g/L: K_2_HPO_4_ 0.225, KH_2_PO_4_ 0.225, NaCl 0.46, (NH_4_)_2_SO_4_ 0.225, MgSO_4_⋅7H_2_O 0.117, NaHCO_3_ 4.2, fumaric acid 4.64; in ml/L: sodium lactate 2.59, trace elements, and vitamin solutions 10 ml each] (Saltikov et al., 2003). These bottles were incubated statically in the dark at 30°C. After 48 h (late-logarithmic phase), the cells were washed twice with anoxic 30 mM bicarbonate buffer by centrifuging at 2,509*g*/30 min, and the resulting pellet was re-suspended in a small volume of the same buffer, keeping the anoxic conditions throughout this washing process. The concentration of cells was measured as OD_600_ in a Jenway 6715 UV/Vis spectrophotometer and then diluted for inoculation into the arsenic-Fe(III)-(oxyhydr)oxide thin film experiments.

### Arsenical Fe(III)-(oxyhydr)oxide Thin Film

Amorphous Fe(III)-(oxyhydr)oxide (ferrihydrite) was prepared by dissolving 16.2 g of FeCl_3_ in 500 ml of deionized water (_*d*_H_2_O), the pH was raised to 7.0 by adding a 10 M NaOH solution under constant stirring and the resulting solution was washed with _*d*_H_2_O and centrifuged (2,688*g*/20 min), with the process repeated six times (Schwertmann and Cornell, 2000). The final concentration of biologically available Fe in the ferrihydrite suspension was 140 mmol per liter slurry. An As(V) solution as sodium arsenate (Na_2_HAsO_4_⋅7H_2_O, Sigma-Aldrich) was prepared and mixed to the ferrihydrite to give a final concentration of 12% mol/mol As/Fe. This mixed solution was stirred on a roller shaker for 24 h to promote the sorption of As(V) on the Fe(III) mineral surface sites. Thin films of arsenical Fe(III)-(oxyhydr)oxide were prepared on boron-doped silicon wafers (7.2 mm × 7.2 mm × 0.5 mm) by pipetting 40 μl of the 12% mol/mol As/Fe solution and allowing to dry overnight.

### Preparation of Microbial Samples for NanoSIMS Analysis

Microbial incubation samples for NanoSIMS analysis were prepared in 15 ml anoxic serum bottles. The thin film-coated Si wafers were placed vertically in a plastic holder that was fixed to the bottom of the bottle with silicon grease. Freshwater medium (in g/L: NaHCO_3_ 2.5, NH_4_Cl 0.25, NaH_2_PO_4_⋅H_2_O 0.6, KCl 0.1, vitamin mix, and mineral mix 10 ml each) (Wilkins et al., 2007) was added to the serum bottles (7 ml) and amended with 20 mM ^13^C-labeled sodium lactate [^13^CH_3_CH(OH)CO_2_Na, Sigma-Aldrich^®^]. In these experiments, the arsenical Fe(III)-(oxyhydr)oxide was the sole terminal electron acceptor, and ^13^C-labeled lactate was used as the electron donor, where ^13^C was expected to be assimilated by the metabolically active cells and so allow their identification during the experiment. Cells of both *S.* ANA-3 strains were inoculated in three replicates under anoxic and sterile conditions to give a final OD_600_ of 0.5, following a procedure reported previously (Newsome et al., 2018). The bottles were incubated at 30°C in the dark for 11 days. Two control experiments were prepared, *S*. ANA-3 WT with no electron donor added (“no donor”), to assess the effect of cells with no metabolic activity, and uninoculated freshwater medium (“no cells”), to assess abiotic reactions.

### Analytical Methods

Supernatants were withdrawn on days 0, 4, 8, and 11 of incubation to measure aqueous Fe(II), As species and flavins. Fe_*total*_ and As_*total*_ were only measured on the last day of incubation. The 0.5 M HCl-extractable Fe(II) and total biologically available Fe(III) were quantified by reacting the aqueous samples with a ferrozine solution and measuring the absorbance at 562 nm, following a standard methodology (Lovley and Phillips, 1986). As(III) and As(V) species in solution were quantified by diluting the samples in _*d*_H_2_O and analyzed by inductively coupled plasma mass spectrometry (ICP-MS, 7500CX; Agilent Technologies, United States). Fe_*total*_ and As_*total*_ were measured by acidifying the aqueous samples in 2% v/v HNO_3_ and analyzed by inductively coupled plasma atomic emission spectroscopy (ICP-AES, Perkin–Elmer Optima 5,300 DV). FMN and riboflavin were measured in the supernatants using HPLC with a fluorescence detector, as detailed elsewhere (Newsome et al., 2018). Riboflavin and FMN solutions (0.1–1.0 μM) were used as references. Plots and statistical analyses were conducted using OriginPro 2019b^®^.

### Sample Preparation for Scanning Electron Microscopy and NanoSIMS Imaging

On day 11, all the Si wafers (samples and controls) were removed from the serum bottles, preserved by chemical fixation with glutaraldehyde and dehydrated sequentially with ethanol in an anaerobic cabinet, as detailed previously by Newsome et al. (2018). The wafers were removed from the last ethanol solution, air dried in an anaerobic cabinet overnight and once dry, coated with 10 nm of Pt using a sputter coater.

### Scanning Electron Microscopy

Scanning electron microscopy (SEM) imaging was used to localize areas of interest with cells and mineral, using a FEI Quanta 650 FEG SEM with a 15 kV beam. The SEM image acquisition was kept to large areas to reduce beam damage to the cells as much as possible. The wafers were stored under anoxic conditions between analyses.

### NanoSIMS Imaging

Two wafers of each sample were analyzed in a NanoSIMS 50 L ion microprobe (CAMECA, France) using a 16 keV Cs^+^ primary ion beam. The primary ion beam was scanned over the surface of the samples with a current of 1.08–0.78 pA to obtain a spatial resolution of ≈400–100 nm (aperture D1 = 3–5). The CAMECA mass resolving power values of 5,500 and 7,000 were used (ES = 3 and AS = 2) for detectors 2 (mass 13) and 7 (mass 75), respectively. Iron metal, gallium arsenide and a clean silicon wafer were used as reference materials to improve mass resolution and avoid peak overlaps from molecular interferences, such as ^12^C^1^H^–^ at mass 13, ^28^Si_2_^16^O^–^ at mass 72, and ^56^Fe^19^F^–^ at mass 75 (as detailed in the next section). The following negative secondary ions were collected simultaneously: ^12^C, ^13^C, ^12^C^14^N, ^28^Si, ^56^Fe^12^C, ^56^Fe^16^O, and ^75^As were analyzed using a double focusing mass spectrometer. Images were collected at a dwell time of 5,000 μs px^–1^ with a pixel resolution of 256 × 256. Additionally, an ion-induced secondary electron (SE) image was obtained. The ^12^C, ^13^C, and ^12^C^14^N signals were used to identify the biomass, and the ^13^C/^12^C ratio was used to identify active cells by their ^13^C accumulation (above the 1.11% natural ^13^C/^12^C abundance); ^28^Si was used to image the wafer surface; ^56^Fe^12^C and ^56^Fe^16^O were used as ^56^Fe proxies because ^56^Fe has a low ionization under Cs^+^ bombardment (Wilson, 1995) [^56^Fe^16^O is easily detected on the Fe(III) oxyhydroxide, whereas ^56^Fe^12^C is easily detected on the cell surface], and along with ^75^As, these secondary ions were used to map the arsenical Fe(III) oxyhydroxide. Implantation of Cs^+^ ions at a dose of 1 × 10^17^ ions cm^–2^ was used to remove the Pt coating and reach steady state on the sample surface before collecting chemical images (McPhail and Dowsett, 2009).

#### Mass Interference at Mass 75

An important mass interference at mass 75 was detected. After performing tests with standards and a non-silicon-based sample matrix (Nunc^TM^ Thermanox^TM^ coverslips; Thermo Fisher Scientific), it was concluded that the mass interference was ^56^Fe^19^F^–^, with ^56^Fe originating from the Fe(III)-(oxyhydr)oxide and ^19^F likely originating from residual vacuum in the NanoSIMS analysis chamber. This mass interference was avoided by improving the mass resolving power to over 7,000 and aligning the detector at the left edge of the peak in the high mass resolution (HMR) scan (Supplementary Figure 1), which compromised the intensity of the ^75^As signal, but avoided the isobaric interference. Following this finding, we recommend that for ^75^As^–^ analysis using NanoSIMS, the interference at mass 75 should always be assessed in samples with abundant Fe.

#### NanoSIMS Single-Cell Depth Profiles

To investigate the distribution of Fe and As in single cells, after collecting large field of view images (30–50 μm width), the raster size was reduced to collect depth profiles from single metabolically active cells (field of view = 3–5 μm width). To avoid uncertainties from any As and Fe re-deposition, cells on the Si wafer more than 3 μm away from arsenical Fe(III)-(oxyhydr)oxide grains were selected for depth profile analysis. The spatial resolution was improved to <100 nm using D1 = 5, and in some cases, L1 was modified to 3,000–7,500 V (a smaller D1 aperture produces a smaller probe diameter and higher voltage in L1 increases the current), pixel sizes were 128 × 128 or 256 × 256 and dwell time varied from 5,000 to 20,000 μs px^–1^. Scanning continued in these depth profiles until the ^12^C or ^12^C^14^N signal dropped, indicating the complete sputtering of the cell, and that chemical information had been collected from the whole organism.

#### NanoSIMS Data Analysis

NanoSIMS images were obtained with L’IMAGE (Larry Nittler, Carnegie Institution of Washington). Hue–saturation–intensity (HSI) maps of ^13^C/^12^C ratios and overlay images were obtained with ImageJ using the OpenMIMS plugin (MIMS, Harvard University; www.nrims.harvard.edu). All files were corrected for a detector dead time of 44 ns. Regions of interest (ROIs) were hand-drawn on every image collected using L’IMAGE. For the mineral ROIs, flat areas away from the edge of the mineral were selected to avoid topographic effects. The counts of the relevant masses analyzed (^12^C, ^13^C, ^12^C^14^N, ^56^Fe^12^C, ^56^Fe^16^O, and ^75^As) were normalized to the current and dose on each day of analysis. Box plots for comparison of NanoSIMS counts and one-way ANOVA tests were generated using OriginPro 2019b^®^.

#### 3D Reconstructions of NanoSIMS Single-Cell Depth Profiles

3D reconstructions of the depth profiles were created with Thermo Scientific^TM^ Avizo^TM^ software 9.7.0. These 3D models allowed the detailed observation of the location of trace As and Fe within the cells, in contrast to the conventional 2D visualization in NanoSIMS stack images. Stack data of ^12^C, ^56^Fe^12^C, ^56^Fe^16^O, and ^75^As were first extracted with ImageJ, saved in the “.raw” format file and loaded into Avizo^TM^. The Z depth was compressed to 15–20%, to remove the spacing between planes generated by the software and obtain 3D models of cells to scale. The ^12^C signal was used to generate the bacteria surface by averaging the counts over 2–3 pixels, then, the “generate surface” and “show surface” commands were used sequentially and the “transparent” display at 80% was selected. The ^56^Fe^12^C, ^56^Fe^16^O, and ^75^As signals were averaged to 1 pixel (because of the lower ion counts) and overlapped on the ^12^C surface by displaying as “points” to enable their visualization.

## Results

### Mobilization of Fe and As to the Aqueous Phase

**FIGURE 1:**
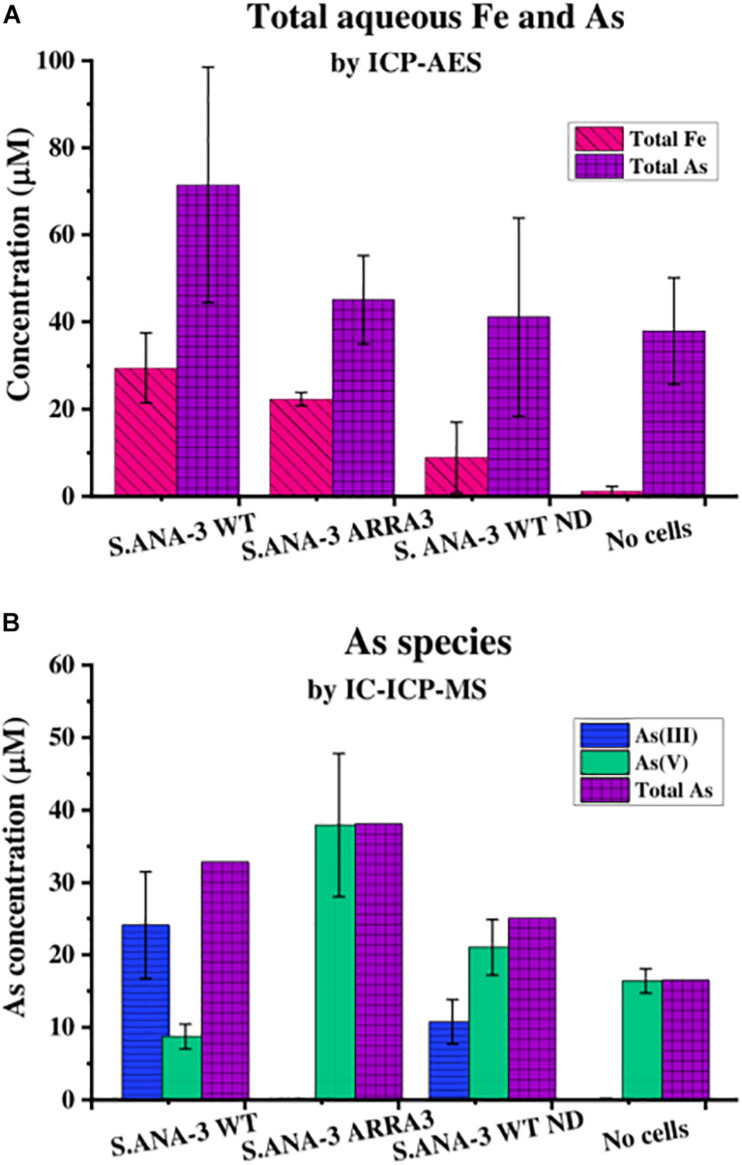
Aqueous geochemistry of the experiments at day 11. **(A)** Total As and Fe in solution quantified by ICP-AES and **(B)** As species in solution quantified by IC-ICP-MS, where total As is the sum of As(III) and As(V) quantified by this method. The Fe solubilized by ^13^C-incubated *S*. ANA-3 WT was significantly higher at the 95% confidence level (*p* < 0.05, Tukey–Kramer test), whereas As solubilization in this sample was not significantly higher than in the other conditions (*p* > 0.05, Tukey–Kramer test). Only the *S*. ANA-3 WT experiments released As(III), including the “no donor” control (*S*. ANA-3 WT ND); however, the solubilization of As(III) was significantly higher at the 95% confidence level (*p* < 0.05, Tukey–Kramer test) in the ^13^C-incubated sample than in the “no donor” control. Results are average of three replicates, and error bars indicate standard deviation.

Measurement of aqueous Fe(II) by ferrozine showed that by day 11, insoluble Fe(III) had been mobilized to soluble Fe(II) by both *S*. ANA-3 strains incubated with ^13^C-lactate as the electron donor (26 μM by the WT and 21 μM by the ARRA3) (Supplementary Figure 2A). No Fe(II) was detected in the “no donor” or in the “no cells” controls (Supplementary Figure 2A). The Fe_*total*_ solubilized at day 11 (29 μM by the WT and 22 μM by the ARRA3) was measured by ICP-AES (Figure 1A), and the values were comparable to the total Fe(II) levels measured by ferrozine. However, ICP-AES also allowed the detection of small quantities of Fe_*total*_ in the “no cells” controls (1 μM) by day 11 (Figure 1A), suggesting that the ferrihydrite thin film was partially soluble under the experimental conditions. Slightly higher Fe_*total*_ levels were measured in the “no donor” control (9 μM), suggesting that the washed cells retained some reducing power. In earlier exploratory work (Supplementary Figure 2B), Fe_*total*_ was only mobilized in incubations with cells and electron donor, and its concentration increased with incubation time.

The As_*total*_ measured by ICP-AES showed that *S.* ANA-3 WT solubilized higher amounts of As (71 μM), although this value was not significantly higher (*p* > 0.05, Tukey–Kramer test) than the ARRA3 mutant (45 μM), *S*. ANA-3 WT “no donor” (41 μM) and the “no cells” control (38 μM) (Figure 1A). The As mobilization observed in the “no cells” control implies that As was partially desorbed under these experimental conditions, as observed previously (Newsome et al., 2018), and consequently, approximately 50% of the As mobilized in the experiments with cells is likely to be due to abiotic desorption. *S*. ANA-3 WT released 83% of the total As in the form of As(III), whereas the ARRA3 cells released negligible (0.2%) As(III), as expected given its lack of an As(V) reductase (Figure 1B and Supplementary Figure 3). The “no donor” control released 33% of As_*total*_ in the form of As(III), which was somewhat surprising, although this again may be explained by the presence of residual endogenous reduced cofactors within the cells at the start of the experiment (Figure 1B and Supplementary Figure 3). It is noteworthy that As(III) levels remained constant from day 4 of incubation in this control experiment, whereas the ^13^C-incubated WT showed an increasing As(III) release throughout the period of incubation. Similarly, solubilized As(V) increased through time in all experiments and controls. The abiotically desorbed As from the “no cells” control was 99.9% As(V) (Figure 1B and Supplementary Figure 3). There were slight discrepancies between the As_*total*_ quantification (Figure 1A) and the total As estimation obtained by adding the two As species (Figure 1B), potentially due to the sample preparation, where the As_*total*_ by ICP-AES samples was extracted in HNO_3_, possibly solubilizing more As, in contrast to the ICP-MS samples that were prepared in _*d*_H_2_O.

### Flavins Secretion

**FIGURE 2:**
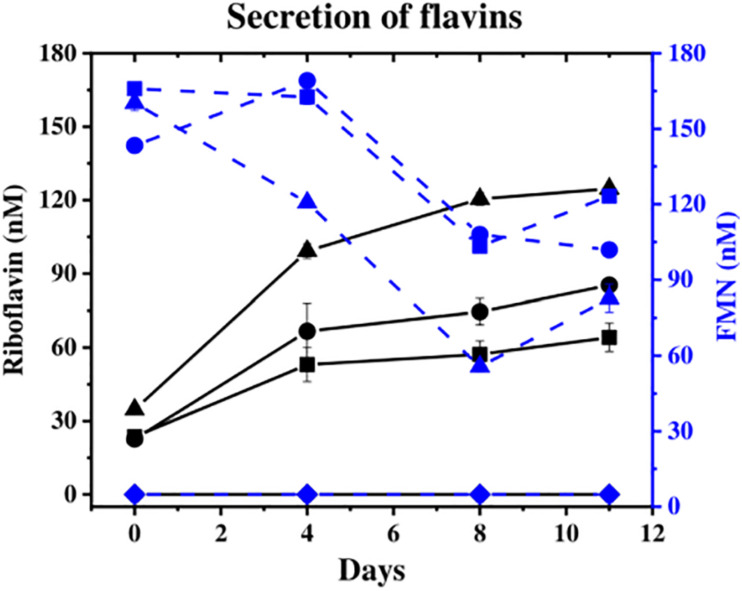
Secretion of flavins during the experiments. Riboflavin is shown in solid black lines and FMN in dashed blue lines [*S*. ANA-3 WT (■), *S*. ANA-3 ARRA3 (∙), *S*. ANA-3 WT no donor (▲) and “no cells” control (◆)]. Results are average of three replicates. Units: nM.

The concentration of flavins (FMN and riboflavin), which can act as extracellular electron shuttles to Fe(III) oxides, was monitored in the aqueous phase. Flavins were detected at similar levels by day 11 in all cultures except in the “no cells” control (Figure 2), where the “no donor” control released 205 nM total flavins, followed by ^13^C-incubated *S.* ANA-3 WT (186 nM) and ARRA3 (185 nM). From the start of the experiment, FMN was detected in the supernatants of all experiments with added cells, and its concentration decreased over the incubation time (Figure 2), whereas riboflavin concentrations increased (Figure 2). Although the original freshwater medium contained 61 nM riboflavin, none was detected in the “no cells” control, suggesting that all the riboflavin detected in the experiments inoculated with *Shewanella* spp. was produced by the cells.

### Localizing Cells in SEM

**FIGURE 3:**
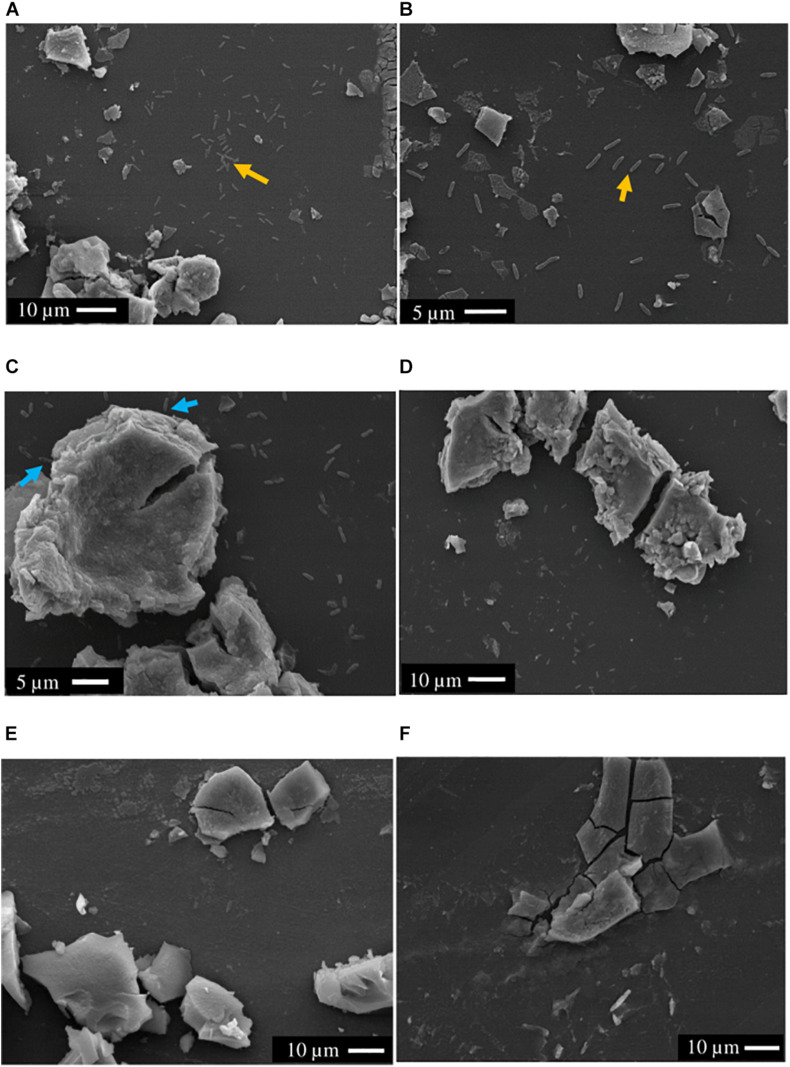
SEM images of axenic cultures and control experiments prior to NanoSIMS. **(A,B)** *S*. ANA-3 WT, **(C,D)** *S*. ANA-3 ARRA3, **(E)** *S*. ANA-3 WT “no donor” and **(F)** “no cells” control. Yellow arrows **(A,B)** indicate examples of cells located on the Si wafer, and cyan arrows **(C)** indicate examples of cells adjoining the Fe(III) mineral. Few cells were observed in the *S*. ANA-3 WT “no donor” control **(E)**.

Scanning electron microscopy showed that the majority of cells (>90%) of both *S.* ANA-3 strains incubated with ^13^C-labeled lactate were observed at distances of more than a micron away (1–20 μm) from the Fe(III) mineral on the Si wafer surface (Figure 3). A small proportion of the cells of both *S.* ANA-3 strains were observed on the Si wafer but adjoining the Fe(III) mineral (Figure 3C). Very few cells were observed to be present directly on the mineral surface, in contrast to previous experiments with *G. sulfurreducens* (Newsome et al., 2018), which is known to require direct contact with insoluble Fe(III) minerals to respire them. Scarce cells were observed in the “no donor” control, suggesting a much lower biomass production than the ^13^C-incubated WT and ARRA3 samples, as expected, due to the lack of an organic substrate to use as electron donor and carbon source (Figure 3E).

### Identifying Active Cells With NanoSIMS

**FIGURE 4:**
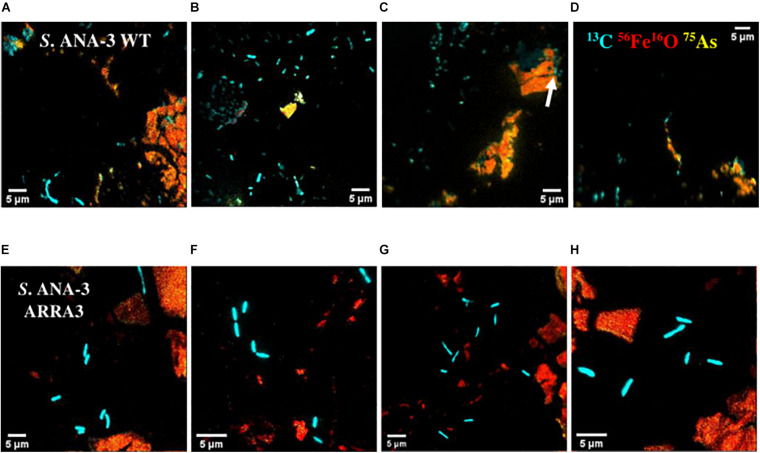
NanoSIMS overlay images of *Shewanella* sp. ANA-3 strains WT **(A–D)** and ARRA3 **(E–H)** incubated with ^13^C-labeled lactate. Cyan is ^13^C, red is ^56^Fe^16^O, and yellow is ^75^As. Notice that most of the cells are on the Si wafer, except for a few WT cells on the Fe(III)-(oxyhydr)oxide mineral surface [white arrow in panel **(C)**].

Not all the cells observed by NanoSIMS accumulated ^13^C, and these were considered to be metabolically inactive. The majority of the active cells of both *S.* ANA-3 strains were observed at a distance from the Fe(III)-(oxyhydr)oxide mineral on the uncoated Si wafer surface of all the areas analyzed for each strain (*N* = 94 for the WT and *N* = 71 for the ARRA3) (Figure 4 and Supplementary Figures 4, 5). A few active cells were observed to be in direct contact with the Fe(III)-(oxyhydr)oxide surface, either on the uncoated Si wafer surface but adjoining the Fe(III)-(oxyhydr)oxide mineral (*N* = 17 for the WT and *N* = 22 for the ARRA3) or growing directly on the Fe(III)-(oxyhydr)oxide surface (*N* = 5, only in the WT) (Figure 4C). The mean ^13^C/^12^C accumulation of *S*. ANA-3 WT was 7.4% ± 5.7 for cells on the uncoated Si wafer, 4.7% ± 1.7 for cells adjoining the mineral and 3.9% ± 0.5 for cells growing on the Fe(III) mineral surface; this implies that overall, the WT cells accumulated 3–6 times more ^13^C than the natural ^13^C/^12^C ratio (Supplementary Figure 6A). The ARRA3 mutant cells accumulated a mean ^13^C/^12^C of 7.1% ± 4.5 for cells on the uncoated wafer and 5.2% ± 0.6 for cells adjoining the Fe(III) mineral, implying an accumulation of up to 4–6 times the natural ^13^C/^12^C ratio (Supplementary Figure 6B). The mean ^13^C/^12^C of cells on the uncoated Si wafer compared to other locations on the sample [adjoining and/or growing directly on the Fe(III) mineral] was not significantly higher (*p* > 0.05, Tukey–Kramer test) in cells of both *S*. ANA-3 strains. Chemical fixation can dilute the ^13^C fraction in cells (Musat et al., 2014), leading to an underestimation of the incorporation rates of isotopically-labeled lactate, although the estimated ^13^C/^12^C ratio was appropriate to locate metabolically active cells and the calculation of absolute ratios was not the aim of this work. The areas analyzed in NanoSIMS can be seen in Supplementary Figures 4, 5 for the WT and ARRA3 strains, respectively.

**FIGURE 5:**
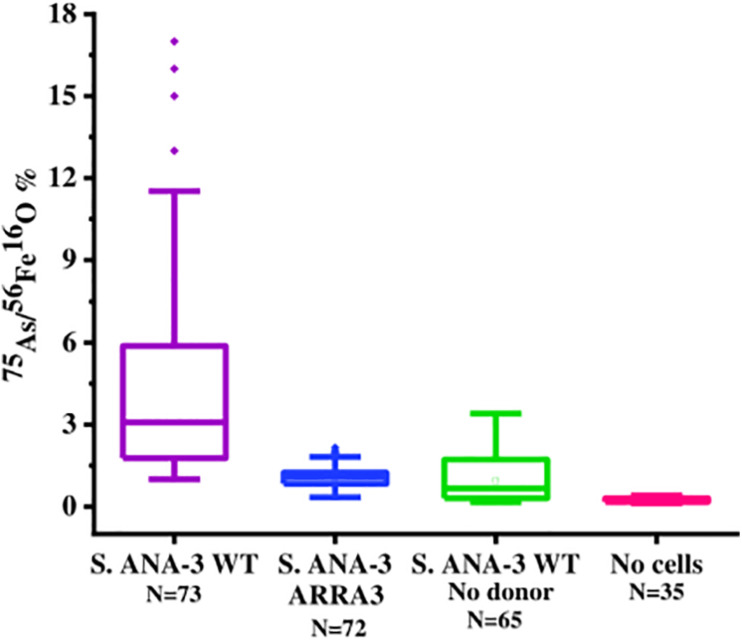
Box plot of ^75^As counts as% of ^56^Fe^16^O counts on areas of the Fe(III)-(oxyhydr)oxide mineral surface in all experiments and controls at day 11 of incubation. The ^75^As/^56^Fe^16^O% in the Fe(III) mineral of *S*. ANA-3 WT was significantly higher at the 99% confidence level (*p* < 0.01, Tukey–Kramer test) than that in the mineral surfaces of the rest of the experiments and controls.

### Mapping As and Fe on the Fe(III)-(oxyhydr)oxide Mineral With NanoSIMS

As and Fe on the Fe(III) mineral surface were mapped using NanoSIMS imaging, where ROIs were selected in all samples to compare normalized ^56^Fe^16^O and ^75^As counts (Supplementary Figure 7). ^75^As and ^56^Fe^16^O showed a positive correlation in all samples, as shown by linear regression fitting, where R^2^ was above 0.6 in all plots (Supplementary Figure 7). The ^75^As counts as a % of ^56^Fe^16^O counts were compared (Figure 5); the mean ^75^As/^56^Fe^16^O ratio was 6.0% ± 6.7 on the Fe mineral of the *S*. ANA-3 WT, 1.1% ± 0.4 in *S*. ANA-3 ARRA3, 0.9% ± 0.8 in the *S*. ANA-3 WT “no donor” and 0.2% ± 0.1 in the “no cells” control. The ^75^As/^56^Fe^16^O ratio on the mineral in the WT sample was significantly higher than that on the mineral areas of the ARRA3 and control samples at the 99% confidence level (*p* < 0.01, Tukey–Kramer test).

### Association of As and Fe With the Cells Revealed by NanoSIMS Single-Cell Depth Profiles

**FIGURE 6:**
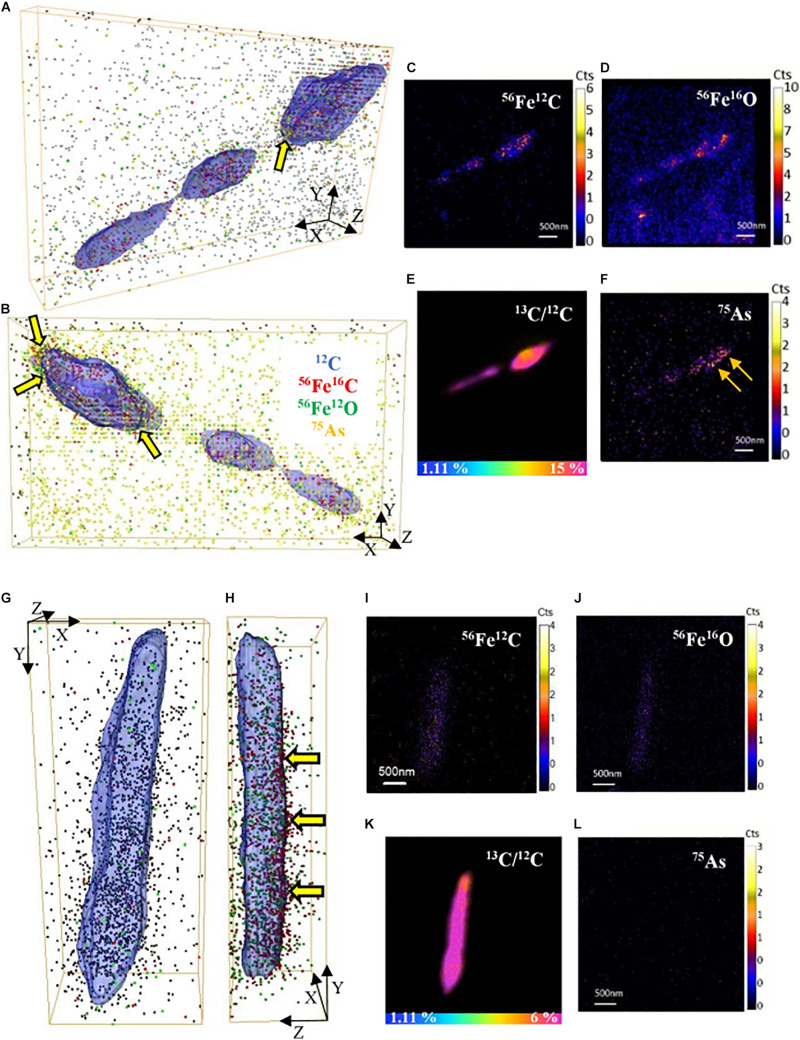
3D reconstructions of single-cell depth profiles of *S*. ANA-3 strain WT **(A–F)** and ARRA3 **(G–L)**. **(A,B,G,H)** are 3D reconstructions showing two angles of the same cell, where ^12^C is displayed in blue (surface), ^56^Fe^12^C as red dots, ^56^Fe^16^O as green dots and ^75^As as orange dots. Panels **(C–F,I–L)** are NanoSIMS stack images (50 planes) of ^56^Fe^12^C, ^56^Fe^16^O, ^75^As and the ^13^C/^12^C% ratio of the cells in panels **(A–B** and **G–H)**, respectively. The horizontal color scale bars in panels **(E,K)** indicate the ^13^C-accumulation percentage. ^56^Fe^12^C and ^56^Fe^16^O counts were imaged in the cells of both strains, and low counts of ^75^As were imaged in the WT strain but not in the ARRA3 strain. The cell in panels **(A–B)** was zoomed in from AOI7 in Supplementary Figure 4 (yellow box); the cell in panels **(G–H)** was zoomed in from AOI9 in Supplementary Figure 5 (yellow box). The yellow arrows in panels **(A**,**B,H)** are pointing at ^75^As and/or ^56^Fe^12^C located at the cell surface.

Fe and As were observed to be co-located with cells in the NanoSIMS analysis using large fields of view (30–50 μm). Therefore, we selected single active cells to collect NanoSIMS depth profiles at higher spatial resolution and prolonged acquisition to image trace Fe and As in active cells of both *S*. ANA-3 strains. On the surface of the cells, low signal of Fe (^56^Fe^12^C and ^56^Fe^16^O) and As (only in the WT cells) was imaged (Figures 6A,B,G,H). Although ^56^Fe^12^C was expected to produce a more intense signal than ^56^Fe^16^O due to the naturally high ^12^C content on the cell surface, the ion counts were similar. For the ARRA3 strain, Fe was observed on the cell surface but not As (Figures 6G,H); in contrast, the WT exhibited Fe and As (Figures 6A,B). The ^56^Fe^16^O and ^75^As counts on the cells were much lower than those on the Fe(III) mineral surfaces (Supplementary Figures 4, 5), where ^56^Fe^16^O and ^75^As counts on the Fe(III) minerals were at least one order of magnitude higher than those on the cells. Additional 3D models of single-cell depth profiles of duplicate cells of both *S*. ANA-3 strains are provided as Supplementary Figure 8.

## Discussion

### Flavins Support Fe(III) Respiration at a Distance but Not As(V) Respiration

NanoSIMS imaging showed that cells of both strains accumulated ^13^C at variable levels above natural abundance, which could reflect, for example, different growth stages, stochastic gene expression, etc. (Peteranderl and Lechene, 2004; Zimmermann et al., 2015). A small number of active WT cells (*N* = 4) were observed on the Fe(III) surface. We hypothesize that these cells directly transferred electrons from *c*-type cytochromes on the cell surface to the Fe(III)-(oxyhydr)oxide mineral, obviating the use of endogenously produced flavins. The majority of active cells (90% of the total) were imaged at a distance from the Fe(III) mineral, suggesting that the dominant Fe(III)-(oxyhydr)oxide reduction mechanism in *S.* ANA-3 is not through direct cell–mineral contact but through electron shuttling under these experimental conditions. This is in agreement with previous observations of *Shewanella* species not requiring direct contact with the Fe(III) mineral that they were respiring, instead using flavins (endogenous or exogenous) to mediate the extracellular transfer of electrons in non-contact scenarios (von Canstein et al., 2008; Edwards et al., 2015; Xu et al., 2016). It has been suggested that the energy available from the reduction of Fe(III) would make it worthwhile for *S*. ANA-3 cells to invest energy in producing and releasing flavins (Covington et al., 2010), which in themselves have high reducing capacities with reduction potentials of −208 and −219 mV for riboflavin and FMN, respectively (van der Zee, 2002). The amount of flavins secreted in all samples in this experiment was in the sub-micromolar level, in accordance to previous reports for other *Shewanella* species (Marsili et al., 2008; von Canstein et al., 2008). Flavins have proven to be effective mediators for reductive Fe(III) dissolution at nanomolar concentrations, and it has been observed that above 1 μM, the rate of Fe(III) dissolution is not enhanced (Wang et al., 2015). Moreover, the conversion of FMN to riboflavin has been reported in cultures of different *Shewanella* strains (von Canstein et al., 2008). In addition to flavins, other molecules acting as electron shuttles could also be at play, such as the elusive and only recently identified ACNQ (2-amino-3-carboxy-1,4-naphthoquinone) (Mevers et al., 2019). The inactive cells imaged by NanoSIMS were most likely present in the starting inoculum and did not actively grow *via* the oxidation of the organic matter analog (^13^C-lactate).

Flavins can catalyze As(III) oxidation in oxic and anoxic conditions (Pi et al., 2019), and it was hypothesized that these redox-active molecules may catalyze As(V) reduction in anoxic systems. However, the demonstrated secretion of flavins yet lack of As(III) production in the ARRA3 experiment suggests that bacterially produced flavins were not involved in As(V) reduction under the conditions tested, and instead only played a role in Fe(III) respiration.

The high concentration of flavins measured in the supernatant of *S*. ANA-3 WT “no donor” control could indicate cell death and lysis. In this control experiment, Fe(II) was undetectable at the end of incubation, confirming that both the electron donor and shuttle are required to complete the transfer of electrons. In natural environments, organic matter typically plays this role, and its availability limits Fe(III) respiration (Shi et al., 2016). Moreover, a percentage of soluble As(V) was reduced to As(III), possibly utilizing the residual intracellular reduced cofactors (for example, NADH, accumulated during biomass production) and indicating that this intracellular respiration mechanism was favored over the extracellular respiration of the insoluble mineral. These results stress the potential role of As(V)-respiring bacteria in As(III) mobilization.

### Dissimilatory Fe(III) Reduction and As(V) Respiration Drive As Mobilization From the Fe(III) Mineral

In these experimental conditions, As was released abiotically, suggesting that these experimental conditions supported As(V) desorption, and if new mineral phases were formed, these did not promote complete As(V) sequestration. In the present work, phosphate was added in higher amounts (3 mM) than typically found in natural environments, to promote bacterial growth, and the high P/As ratio also potentially contributed to abiotic As(V) desorption (Biswas et al., 2014). Given the characteristics of the samples, it was not possible to recover and analyze (unaltered) the low amounts of secondary minerals produced (< 30 mg).

The ^75^As/^56^Fe^16^O% was a useful estimation to investigate the fate of these ions on the solid mineral surface; however, quantification is not straightforward using NanoSIMS, where different artifacts affect the secondary ion yield (Hoppe et al., 2013). Under these experimental conditions, sorption and desorption phenomena took place, along with potential secondary mineralization, which challenge the quantitative interpretation of these results. For these reasons, we are cautious with this data interpretation and only attempt to explain these observations in relation to the complementary aqueous phase observations. The significantly higher ^75^As/^56^Fe^16^O% on mineral areas of ^13^C-incubated *S.* ANA-3 WT (Figure 5), compared to the results in strain ARRA3 and the control experiments, suggests an active mobilization of both As and Fe from the mineral surface by the WT cells, whereas lower ^75^As/^56^Fe^16^O% could suggest preferential As mobilization, driven by Fe(III) reduction and abiotic desorption. This agrees with the higher As_*Total*_ and Fe_*Total*_ mobilized to the aqueous phase in the ^13^C-incubated WT sample (Figure 1). The lower ^75^As/^56^Fe^16^O% observed in samples where only or mostly As(V) was solubilized was previously observed in the local environment of the non-As(V) reducer *G. sulfurreducens* (Newsome et al., 2018). These results could also indicate an As(III) sequestration–precipitation effect by interaction with Fe(II) and/or organic matter (Kocar et al., 2006; Liu et al., 2020), which were only present in the WT sample, a finding that was similarly observed in incubations with other *Shewanella* strains (Jiang et al., 2013; Lee and Hur, 2014). This is contrary to the expectation that solubilized As(V) would re-sorb or precipitate more readily on the Fe(III) mineral, where additionally, competitive sorption could take place under these relatively high phosphate conditions (Zhu et al., 2011; Biswas et al., 2014).

Moreover, these NanoSIMS results contrast with the aqueous phase geochemistry results, where both *S.* ANA-3 strains solubilized As(III) and/or As(V), which remained in solution through the incubation period analyzed. However, the NanoSIMS assessment is limited to the analysis of small regions (1–3 μm^2^) on the solid mineral, which only describes this phenomenon at the microenvironment of cells and is complementary to what is being observed in the bulk surrounding aqueous environment. Our findings suggest that, overall, the Fe(III)-(oxyhydr)oxide [and any post-reduction secondary Fe(II)-containing minerals] had insufficient sites to support As(III) and/or As(V) re-sorption, which may be explained by the low Fe(III) mineral surface compared to the large volume of aqueous medium, where these experimental conditions were designed to mimic the natural groundwater environment.

The advantages of being able to respire both As(V) and Fe(III) were demonstrated by enhanced As and Fe mobilization in both the aqueous and solid phases in the *S*. ANA-3 WT compared to ARRA3, implying that As(V)-respirers, which are frequently Fe(III)-respirers as well (Reyes et al., 2008), may play a key role in As mobilization in sedimentary/anoxic environments.

### Fe and As Associate With the Cell Surface but Are Not Accumulated Intracellularly

A higher signal of surface Fe and As was detected through NanoSIMS single-cell depth profiles, in contrast to intracellular Fe and As (Figure 6 and Supplementary Figure 8). This suggests preferential Fe and As accumulation at the cell surface, which is likely in a medium with soluble Fe(II) and As(III) (Shiraishi et al., 2018), although NanoSIMS does not discriminate the valence of the elements analyzed. In one study, *Shewanella putrefaciens* strain CN32 accumulated iron in the form of intracellular granules (mixed Fe valence) when growing with Fe(III) oxyhydroxides as terminal electron acceptors, but this is the only *Shewanella* strain (and indeed only study) to show these granules (Glasauer et al., 2007). In this work, *S*. ANA-3 did not contain any structures that resembled intracellular Fe granules, rather, the Fe counts imaged in NanoSIMS were scattered. Thus, the ^56^Fe^12^C and ^56^Fe^16^O signals detected on the cell surface of both Fe(III)-respiring strains could have originated from the membrane-bound *c*-type cytochromes that are active during this electron transfer process. Only the WT strain showed ^75^As on the cells, where As(III) was detected as the predominant aqueous As species. We hypothesize that the ^75^As imaged in the WT could be As(III/V) located at the periplasm, where As(V) respiration takes place. The exact accumulation mechanism of As at the cell surface in the WT is unknown; however, As(III) has a high affinity for thiol groups in proteins and can bind to lipids, carbohydrates, amines, amides and aromatic groups on the cell wall (Shen et al., 2013). This suggests that in reducing conditions, solubilized As(III) will likely bind to more sites than anticipated, including organic/biological surfaces, which may deter its mobilization.

### As(V) Is Respired After Being Mobilized to the Aqueous Phase

We identified two possible mechanisms for insoluble Fe(III) reduction by *S.* ANA-3 in our experimental system; most cells were found at a distance from the Fe(III)-(oxyhydr)oxide, with Fe(III) reduction most likely mediated by flavins acting as electron shuttles, whereas a small proportion of cells grew in direct contact with the Fe(III) mineral. Our aqueous geochemistry results showed that As(III) was solubilized throughout the course of the experiment by the WT strain (Supplementary Figure 3A), and complementary early development work showed a similar trend for Fe solubilization measured by ICP-AES (Supplementary Figure 2B) in the ^13^C-incubated samples. This indicates that As(V) respiration did not precede dissimilatory Fe(III) reduction. Moreover, *S*. ANA-3, like other dissimilatory As(V)-respiring bacteria, has a respiratory As(V) reductase located in the periplasm, and this would make the direct reduction of As(V) sorbed to solid Fe(III)-(oxyhydr)oxides unlikely. Our findings indicate that the majority of *S.* ANA-3 WT cells did not directly contact the solid phase As(V), that was sorbed to the Fe(III)-(oxyhydr)oxide, and therefore the reduction of As(V) is most likely to have occurred once it was mobilized to the aqueous phase. This was in accordance with data from cultures of a *Clostridium* strain observed to reduce only soluble As(V) (possibly as a detoxification mechanism) (Langner and Inskeep, 2000) and another study where *Alkaliphilus oremlandii* OhILAs only respired soluble As(V) (Tian et al., 2015).

Even though the characterization of the spatial and temporal fluctuations of As in impacted ecosystems is challenging, we propose a multistep Fe(III) and As(V) reduction mechanism by *S*. ANA-3 in the conditions studied; (i) the cells first reduce the insoluble Fe(III)-(oxyhydr)oxide, mainly through flavins used as electron shuttles, (ii) this reduction leads to the solubilization of Fe(II) and desorption of As(V), (iii) once in solution, As(V) traverses the outer membrane (for example, through porins) and is reduced to As(III) in the periplasm and finally, (iv) some As(III) binds to cells (observed as ^75^As in 3D reconstructions of the WT, but not present in ARRA3), although most of it diffuses from the cells and accumulates in the aqueous phase. As(V) respiration led to the release of As(III); however, Fe(III) reduction was a prerequisite for As(V) respiration; thus, Fe(III) reduction was the controlling mechanism of As mobilization under the conditions tested in this work. We propose that this model of Fe(III)-(oxyhydr)oxide reduction followed by As mobilization could explain the mechanism of As release in reducing aquifers, supported, for example, by recent field data (Gnanaprakasam et al., 2017; Mihajlov et al., 2020). The geochemical conditions in As-impacted sites dictate the fate of arsenic. As(III) will likely persist at lower aquifer depths and under reducing conditions, as there are fewer As sorption sites available, for example, due to lower loadings of Fe(III) minerals, unless As(III)-oxidizing bacteria or minerals with high sorption properties are present (Gorny et al., 2015).

## Conclusion

Under the conditions tested, *S*. ANA-3 cells actively reduced Fe(III)-(oxyhydr)oxide and consequentially desorbed As(V); this As(V) was taken up by the cells from the aqueous phase and respired, producing As(III). The accumulation and persistence of As(III) is key in the toxicity of groundwater destined for human consumption, where Fe(III) and As(V)-respiring bacteria are thought to play a significant role by actively desorbing As(V) and maintaining As(III) levels in the groundwater.

In the present work, NanoSIMS coupled to stable isotope labeling of active cells delivered unique spatial profiles at subcellular resolution of the cells actively respiring iron and arsenic. Imaging this close association between bacteria and respired metals contributes to the understanding of how these microorganisms interact with these electron acceptors and ultimately predict the fate of As (and potentially other metals) in subsurface environments. Interestingly, NanoSIMS allowed the identification of As(III) accumulation at the cell surface, through a retention mechanism that warrants further investigation. Thus, our results also show the capabilities of the technique to image low concentrations of key metals (and other trace elements) at submicron scales, facilitating the identification of cellular location.

Further studies that focus on stimulating microorganisms with ^13^C-labeled organics, supplied to more complex sediment systems collected from As-impacted aquifers, are necessary. This work could potentially be supported using the NanoSIMS approach outlined here, in combination with more traditional microbial imaging techniques, and in due course multi-omic approaches (utilizing stable isotope probing where possible) to identify the causative organisms. This holistic approach could also be applied to a wider range of biogeochemical cycles, which are also involved in controlling the fate of toxic metals in the subsurface.

## Data Availability Statement

The original contributions presented in the study are included in the article/Supplementary Material, further inquiries can be directed to the corresponding author/s.

## Author Contributions

RLA, LN, and JL designed the experiments. RLA carried out the experiments and performed the geochemical, SEM and NanoSIMS data collection and analysis. KM and IL supported the collection of the NanoSIMS data. RLA wrote the manuscript. IL, KM, LN, and JL revised the manuscript. All authors contributed to the article and approved the submitted version.

## Conflict of Interest

The authors declare that the research was conducted in the absence of any commercial or financial relationships that could be construed as a potential conflict of interest.
