# Supplementary material for: Dissimilatory Fe(III) Reduction Controls on Arsenic Mobilization: A Combined Biogeochemical and NanoSIMS Imaging Approach
Source: Front Microbiol. 2021 Feb 22;12:640734. doi: 10.3389/fmicb.2021.640734 (PMC7938665; doi:10.3389/fmicb.2021.640734)
Supplement: Supplementary file 1 [file Data_Sheet_1.PDF]

Supplementary Material

1 Supplementary Figures

NanoSIMS HMR spectrum at mass 75

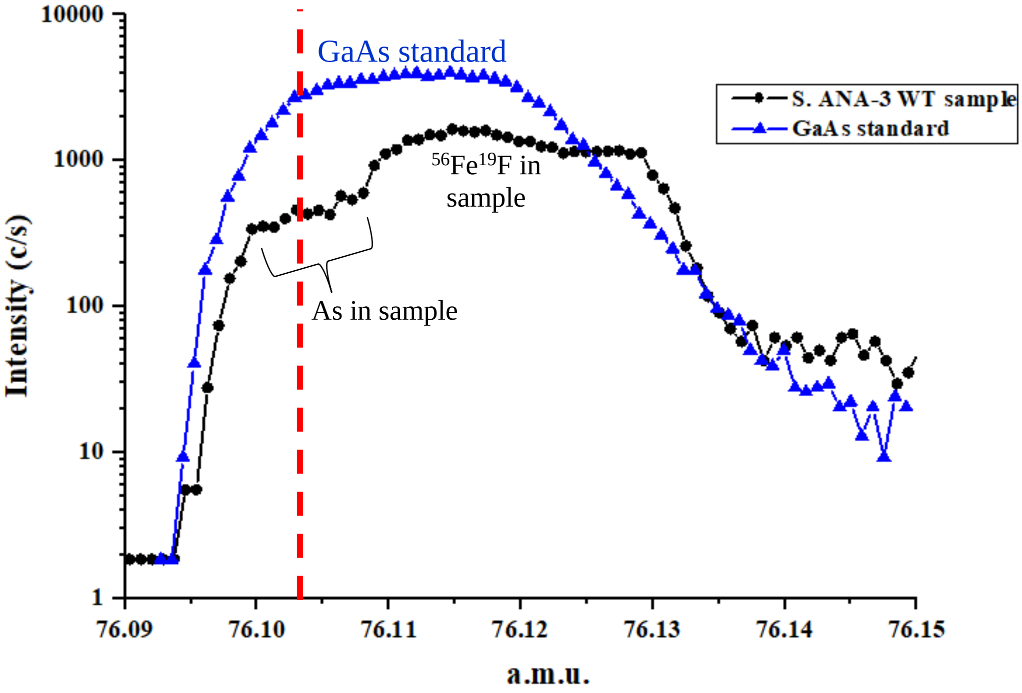

Supplementary Figure 1. NanoSIMS high mass resolution (HMR) spectrum showing the superposition of the GaAs standard peak (▲) and the *Shewanella* ANA-3 WT sample peak (●) after mass resolution was improved (mass resolving power=7330). The red dashed vertical line is where the detector was set after the mass interference experiments. Note that the detector is not set on the flat part of the GaAs standard peak but on the edge of the peak, compromising the intensity of As counts. Note the log scale on the Y axis.

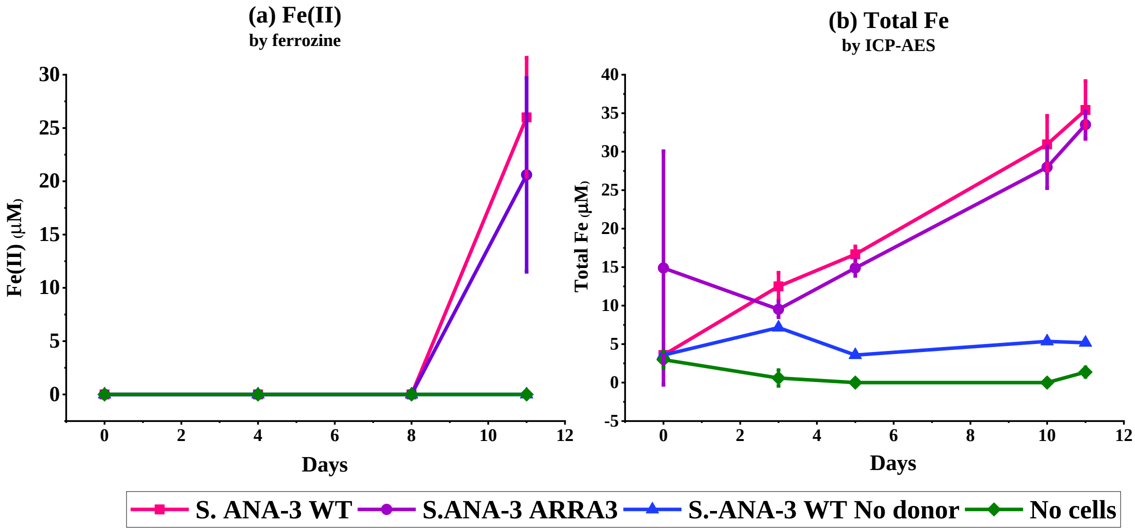

Supplementary Figure 2. Aqueous iron monitored through incubation time, measured as (a) Fe(II) via the ferrozine method, and (b) total Fe measured by ICP-AES. Plot (b) was obtained from earlier exploratory work in our research group. In these experiments only the incubations with cells and electron donor solubilised Fe and Fe(II), as expected. Notice the difference in Fe and Fe(II) measured by the different analytical techniques, where ferrozine is limited in the early quantification of Fe(II), possibly due to its detection limit. Bars represent the standard deviation of three replicates.

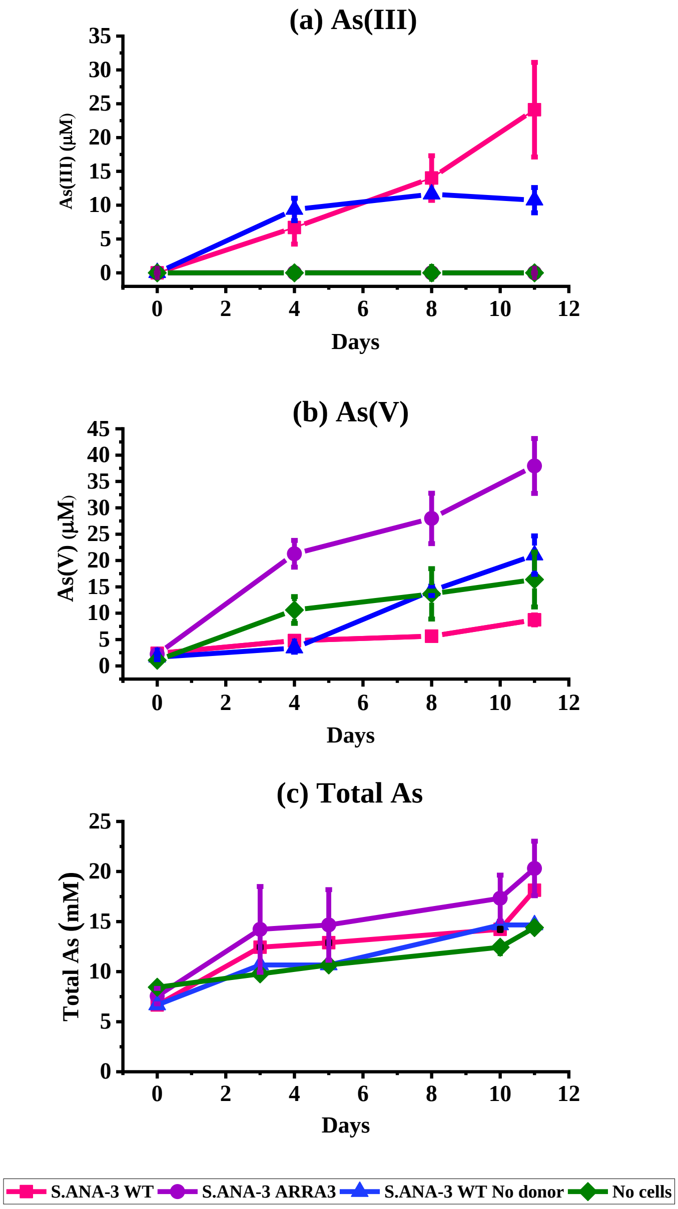

Supplementary Figure 3. Aqueous arsenic monitored through incubation time. (a) As(III), (b) As(V) (both As species measured by ICP-MS), and (c) total As measured by ICP-AES. Plot (c) was obtained from earlier exploratory work in our research group. Notice the lower total As quantified in a previous experiment (plot c) in comparison to the As species plotted in (a) and (b) from these experiment, although the tendency of increasing arsenic release through time remains in the conditions tested. Only the *S*.ANA-3 WT incubations released As(III), including the “no donor” control. Bars represent the standard deviation of three replicates.

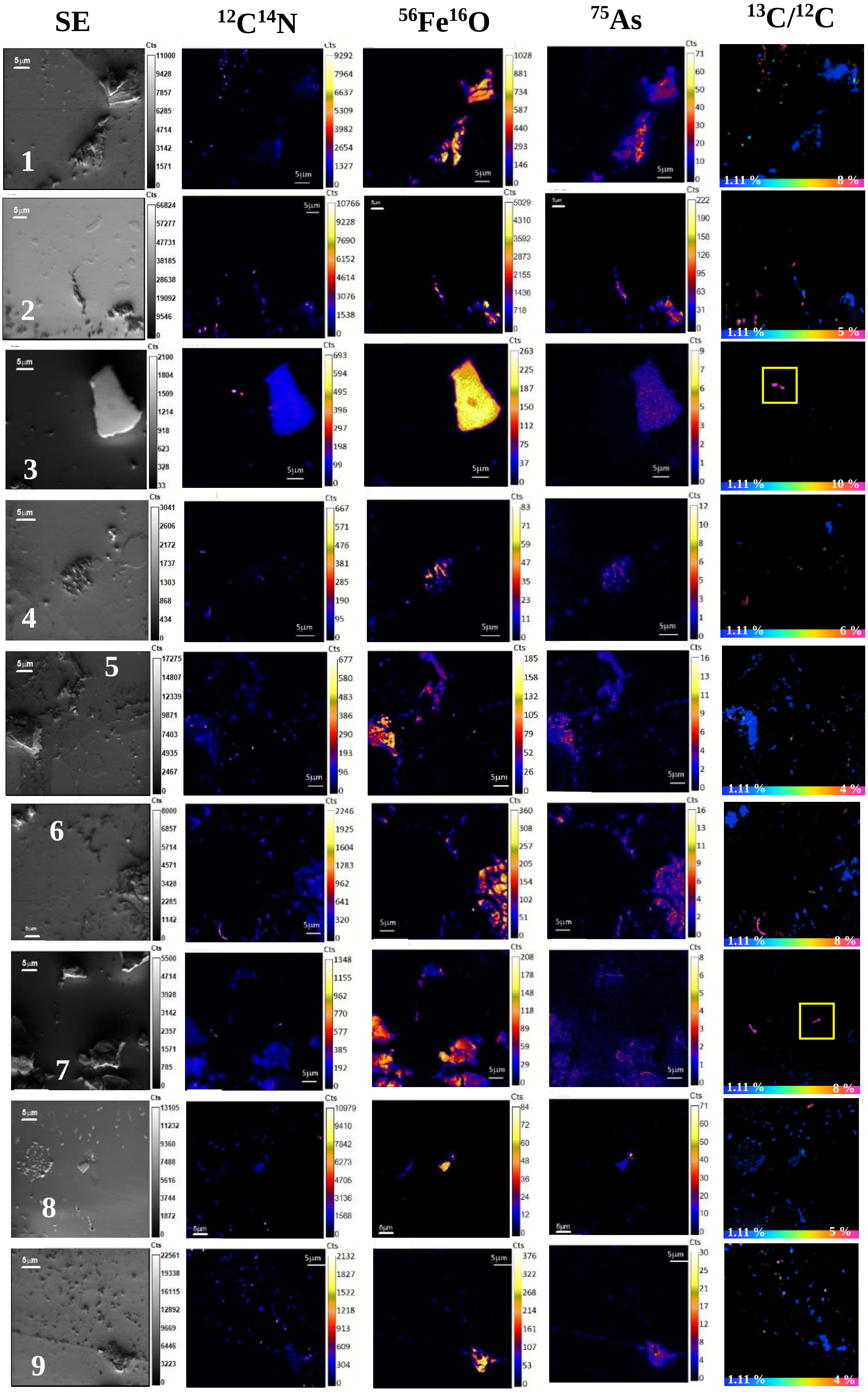

Supplementary Figure 4. Areas of interest (AOI) of *S.* ANA-3 WT analysed in NanoSIMS. The first panel are the secondary electron (SE) images, followed by $^{12}C^{14}N$, $^{56}Fe^{16}O$, ^{75}As and the $^{13}C/^{12}C$ ratio (%). The yellow squares in AOIs 3 and 7 are the cells selected for depth profile and shown in Supplementary Figure 8A and Figure 6A, respectively. Panels 6, 8, 1 and 2 correspond to panels A-D in Figure 4, respectively.

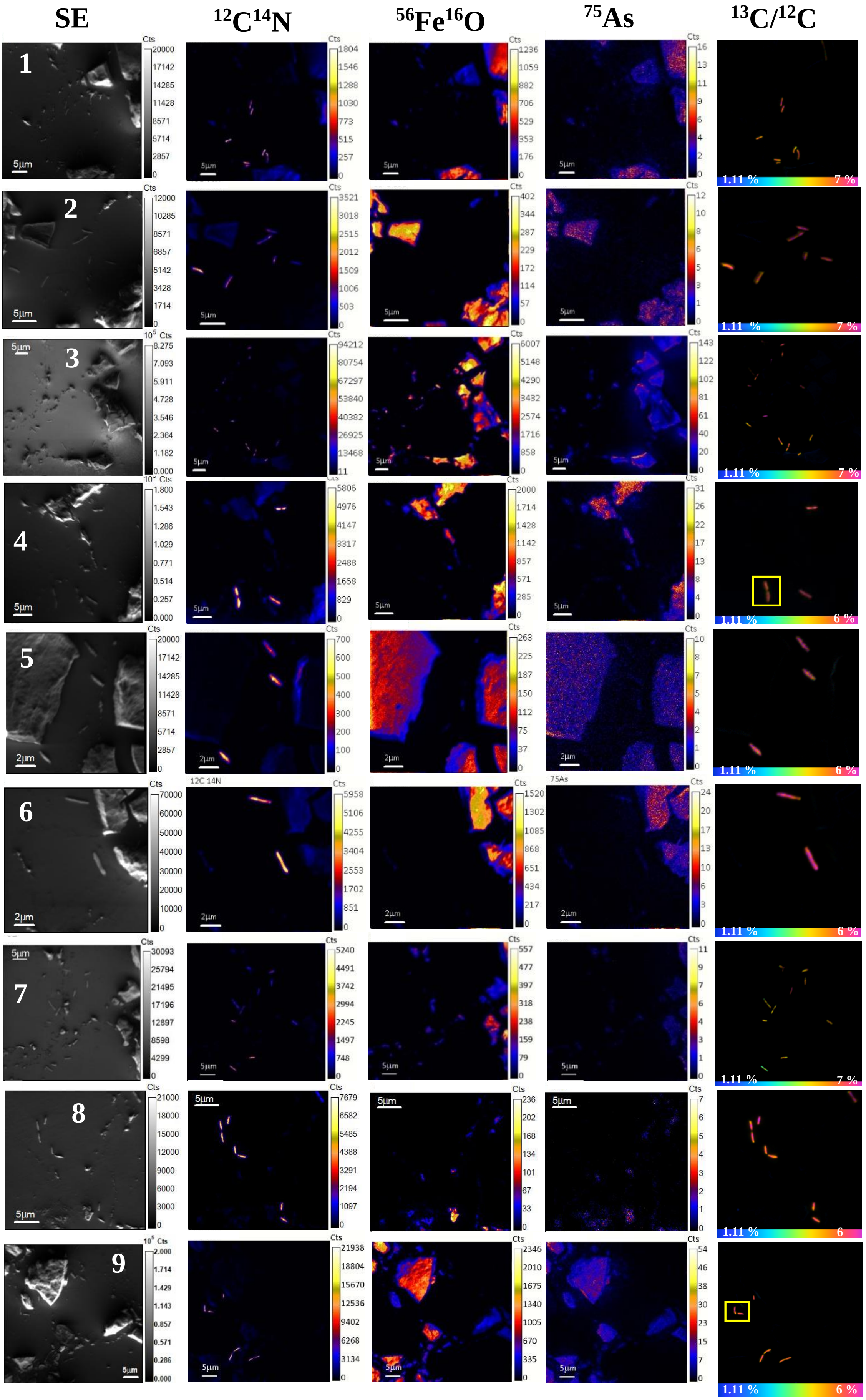

Supplementary Figure 5. AOIs of *S*. ANA-3 ARRA3 analysed in NanoSIMS. The first panel are the secondary electron (SE) images, followed by $^{12}C^{14}N$, $^{56}Fe^{16}O$, ^{75}As and the $^{13}C/^{12}C$ ratio (%). The yellow squares in AOIs 4 and 9 are the cells selected for depth profile and shown in Supplementary Figure 8G and Figure 6G, respectively. Panels 1, 8, 7 and 2 correspond to panels E-H in Figure 4, respectively.

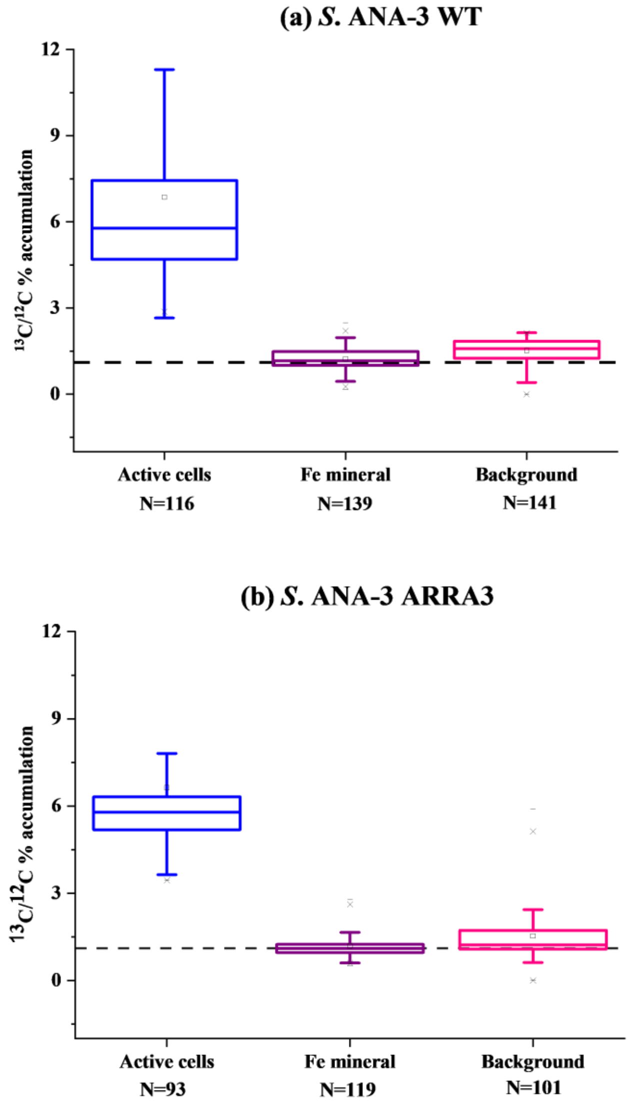

Supplementary Figure 6. Box and whisker plots of the $^{13}C/^{12}C$ ratio in metabolically active cells, Fe(III) mineral and natural background, for comparison. (a) *S*. ANA-3 WT and (b) *S*. ANA-3 ARRA3. The black dashed line is the 1.11 % $^{13}C/^{12}C$ natural abundance.

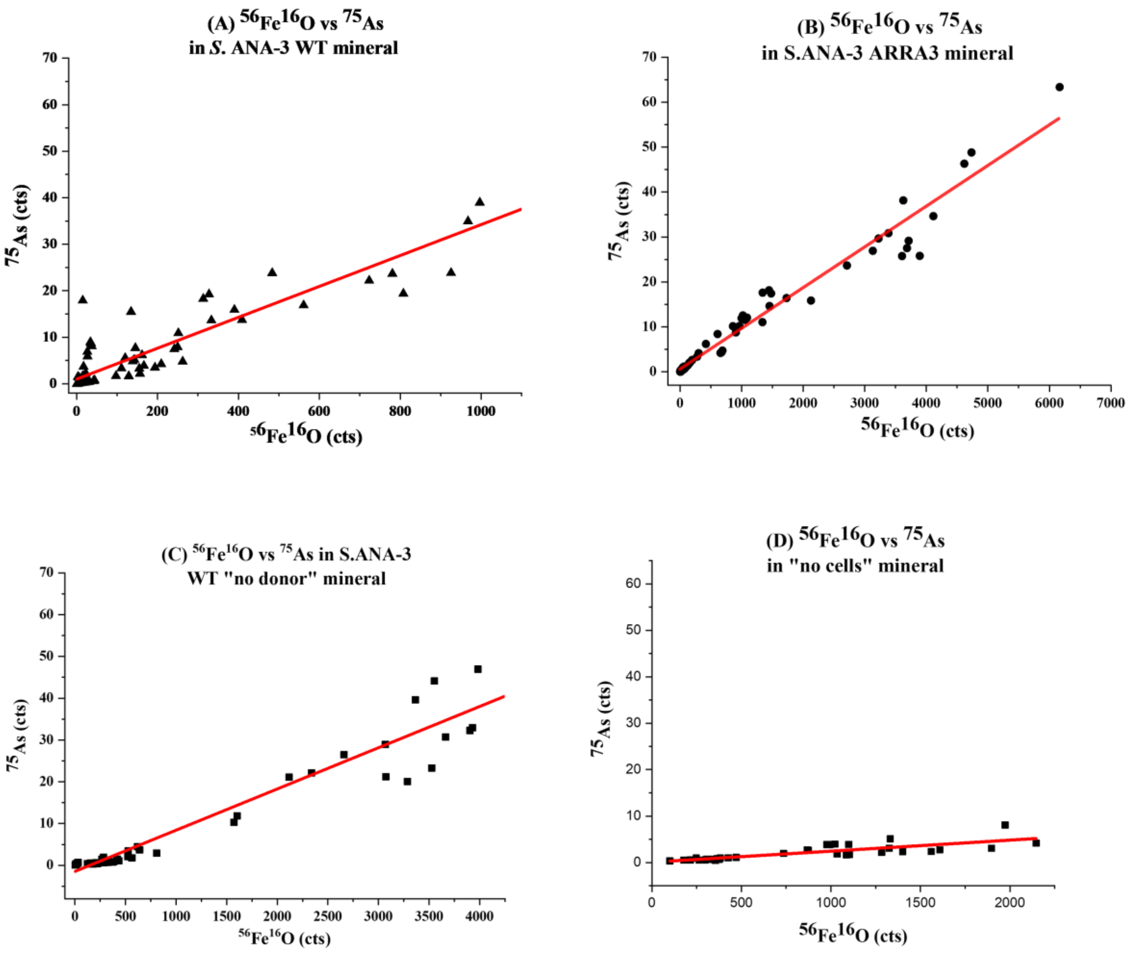

Supplementary Figure 7. Scatter plots of NanoSIMS $^{56}Fe^{16}O$ vs ^{75}As normalised counts on Fe(III) mineral areas of (A) ^{13}C-incubated *S.* ANA-3 WT, (B) ^{13}C-incubated *S.* ANA-3 ARRA3, (C) *S.* ANA-3 WT “no donor” and (D) “no cells” control. The R^2 values are: (A) 0.92, (B) 0.96, (C) 0.94 and (D) 0.64.

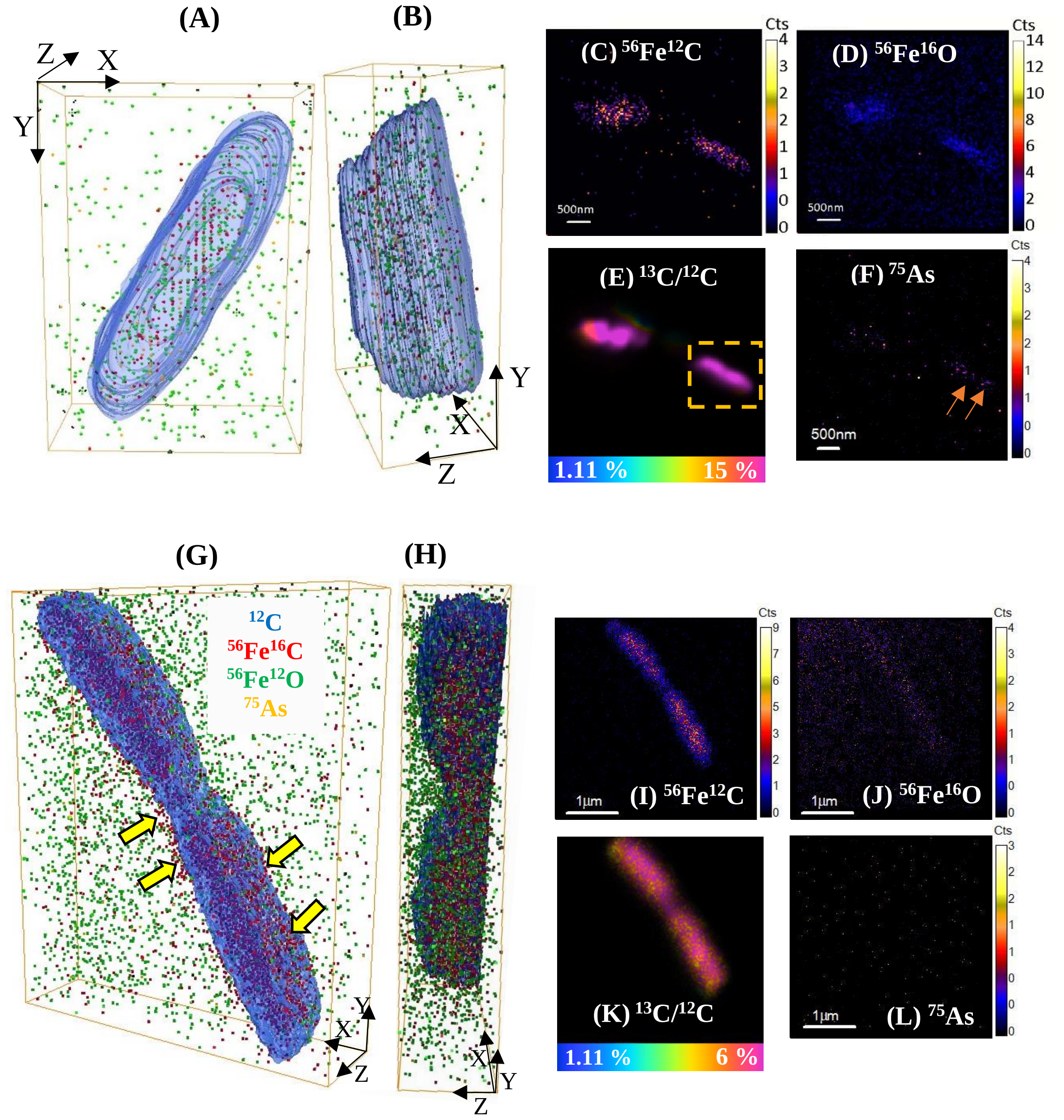

Supplementary Figure 8. Additional 3D reconstructions of single-cell depth profiles of *S.* ANA-3 strain WT (A-F) and ARRA3 (G-L). (A-B) and (G-H) are the 3D reconstructions in two angles, where ^{12}C is displayed in blue (surface), $^{56}Fe^{12}C$ is shown as red dots, $^{56}Fe^{16}O$ as green dots and ^{75}As as orange dots. (C-F) and (I-L) are NanoSIMS stack images of $^{56}Fe^{16}O$, $^{56}Fe^{12}C$, ^{75}As and the $^{13}C/^{12}C$ ratio of the same cells. (C-F) are stacks of 95 planes; (I-L) are stacks of 100 planes. The mustard dashed square in (E) is the 3D modelled cell in (A-B). The horizontal colour scale bars in (E) and (K) indicate the ^{13}C-accumulation percentage. $^{56}Fe^{12}C$ and $^{56}Fe^{16}O$ counts were imaged in the cells of both strains, low counts of ^{75}As were imaged in the WT strain but not in the ARRA3 strain. The cell in panels (A-B) was zoomed-in from AOI3 in Supplementary Figure 4 (yellow box); the cell in panels (G-H) was zoomed-in from AOI4 in Supplementary Figure 5 (yellow box). The yellow arrows in (G) are pointing at surface $^{56}Fe^{12}C$.
